# DEB TACE for Intermediate and advanced HCC – Initial Experience in a Brazilian Cancer Center

**DOI:** 10.1186/s40644-017-0108-6

**Published:** 2017-02-06

**Authors:** Jose Hugo Mendes Luz, Paula M. Luz, Henrique S. Martin, Hugo R. Gouveia, Raphal Braz Levigard, Felipe Diniz Nogueira, Bernardo Caetano Rodrigues, Tiago Nepomuceno de Miranda, Marcelo Henrique Mamede

**Affiliations:** 1 Department of Interventional Radiology, Radiology Division, National Cancer Institute, INCA, Praça Cruz Vermelha 23, Centro, Rio de Janeiro, CEP 20230-130 Brazil; 2 National Institute of Infectious Disease Evandro Chagas, Oswaldo Cruz Foundation, Avenida Brasil 4365, Manguinhos, Rio de Janeiro, 21040-360 Brazil; 3 Department of Interventional Radiology, Radiology Division, Hospital Federal de Bonsucesso, Avenida Londres, 616, Bonsucesso, Rio de Janeiro, 21041-030 Brazil; 4 Department of Interventional Radiology, Radiology Division, Hospital Federal de Ipanema, Rua Antônio Parreiras, 67, Ipanema, Rio de Janeiro, 22411-020 Brazil; 5 Department of Anatomy and Radiology, Full Professor, Medicine School – UFMG, Avenida Presidente Antônio Carlos, 6627 Pampulha, Belo Horizonte, Minas Gerais 31270-901 Brazil

## Abstract

**Background:**

According to Barcelona Clinic Liver Cancer classification transarterial chemoembolization is indicated in patients with Hepatocellular Carcinoma in the intermediate stage. Drug-eluting microspheres can absorb and release the chemotherapeutic agent slowly for 14 days after its intra-arterial administration. This type of transarterial chemoembolization approach appears to provide at least equivalent effectiveness with less toxicity.

**Methods:**

This is a prospective, single-center study, which evaluated 21 patients with intermediate and advanced hepatocellular carcinoma who underwent transarterial chemoembolization with drug-eluting microspheres. The follow up period was 2 years. Inclusion criteria was Child-Pugh A or B liver disease patients, intermediate or advanced hepatocellular carcinoma and performance status equal or below 2. Transarterial chemoembolization with drug-eluting microspheres was performed at 2-month intervals during the first two sessions. The third and subsequent sessions were performed according to the image findings on follow-up, on a “demand schedule”. Tumor response and time to progression were evaluated along the two-year follow up period.

**Results:**

Of the 21 patients 90% presented with liver cirrhosis, 62% had Barcelona Clinic Liver Cancer stage B and 38% had Barcelona Clinic Liver Cancer stage C hepatocellular carcinoma. Average tumor size was 6.9 cm. The average number of Transarterial chemoembolization with drug-eluting microspheres procedures was 3 with a total of 64 sessions. The predominant toxicity was mild. Liver function was not significantly affected in most patients. Two deaths occurred within 90 days after Transarterial chemoembolization with drug-eluting microspheres (ischemic hepatitis and hydropic decompensation). Technical success was achieved in 63 of 64 procedures. The mean hospital stay was 1.5 days. The progression free and overall survival at 1 and 2 years were 73.0% and 37.1%, 73.7% and 41.6%, respectively.

**Conclusion:**

Transarterial chemoembolization with drug-eluting microspheres is able to deliver significant tumor response and progression free survival rate with acceptable toxicity. Larger studies are needed to identify exactly which subset of advanced hepatocellular patients may benefit from this treatment.

**Trial registration:**

study ID ISRCTN16295622. Registered October 14th 2016. Retrospectively registered.

Website registration: http://www.isrctn.com/ISRCTN16295622

## Background

Hepatocellular carcinoma (HCC) has become one of the most common tumors worldwide with approximately 500,000 new cases per year [1]. The main risk factors are infection with hepatitis B and C viruses. Approximately 1.4 to 2.5% of cirrhotic patients with hepatitis C and 1.5 to 6.6% of patients with hepatitis B viruses develop HCC [1]. Other risk factors are toxins (alcohol and aflatoxin B1), metabolic disorders (hemochromatosis, alpha 1-antitrypsin deficiency, cutaneous porphyria, etc.), anabolic steroids consumption and other causes of cirrhosis [1]. One of the most frequently used staging criteria for HCC is the BCLC algorithm (Barcelona Clinic Liver Cancer Staging Classification). Patients are classified accordingly to tumor size, number of hepatic tumors, PS (Karnofsky performance status scale), vascular invasion and extrahepatic spread. Those with a PS of zero, a single tumor larger than 5 cm or three tumors larger than 3 cm without vascular invasion or extra-hepatic disease are classified as intermediate HCC (BCLC stage B) and TACE is the treatment of choice. Patients with PS equal or greater than 1 and/or with portal invasion and/or extrahepatic disease are classified as advanced HCC (BCLC stage C) and Sorafenib was recently approved for the treatment of this subset of patients [2]. Those with advanced liver cirrhosis or PS greater than 2 are classified as terminals (BCLC stage D) and receive supportive therapy [3–5]. Treatments for patients allocated in BCLC stage B and C are considered palliative, differing from surgery, ablation and transplant, the therapeutics options for BCLC stage A HCC, which are recognized curative treatments.

Developed in the last decade, “drug eluting beads” (DEB) for TACE, made with superabsorbent polymers, have the property of absorbing the chemotherapy and slowly release it over several days (up to 14 days) in a steadily sustained manner. In conventional TACE there is a peak on the bloodstream of the chemotherapeutic agent right after the procedure [6, 7]. With DEB TACE there is a slow release of the chemotherapeutic agent into the hepatic tumor, limiting the systemic exposure of the drug and thus potentially reducing the occurrence of side effects. Another important change with this new approach is that TACE protocols are now standardized (diluting instructions are designed by the manufacturers [6]). One of the DEB TACE devices available is DC-Beads® which are precisely calibrated microspheres that are capable of absorbing chemotherapy (eg. doxorubicin). After its administration in liver tumor by intra-arterial injection, these microspheres begin to slowly release chemotherapy in a controlled and sustained manner for 14 days [7]. Experimental studies have shown that TACE with DEB has a secure pharmacokinetic profile and determines effective tumor destruction in animal models [8, 9]. With DEB TACE, chemotherapy plasma concentration is maintained low and constant throughout 14 days. In addition, the chemotherapy agent is maintained longer in contact with the tumor in the case of DEB TACE, but in the conventional technique chemotherapy is quickly eliminated from the liver [9–13].

## Methods

This is a prospective non-randomized study where 21 patients with intermediate and advanced HCC, from a tertiary referral cancer center, were selected and submitted to DEB TACE loaded with doxorubicin from September 2009 thru April 2010. Our primary endpoint of interest was tumor response and progression-free survival and the secondary endpoint was to evaluate the occurrence of adverse events. The DEB TACE procedures were done at 2-month intervals during the first two sessions. From this point on new DEB TACE sessions were performed on demand accordingly to response in magnetic resonance (MR) and clinical outcome. Tumor response was evaluated with liver dedicated dynamic-enhanced MR of the abdomen and interpreted by body-imaging radiologists. Patients unable to perform MR were schedule to undergo computed tomography (CT). Clinical and laboratory tests were performed before and after each session and during hospitalizations, targeting the evaluation of the toxicity and quantification of adverse effects. For the inclusion criteria patients had to be 18 years old or above, present a Child-Pugh A or B (Child-Pugh Classification) status, a PS equal or less than 2, a liver tumor compatible with a BCLC stage B or C HCC which had not been previously submitted to TACE or any intra-arterial treatment.

### TACE with DEB – the procedure

**Fig. 1:**
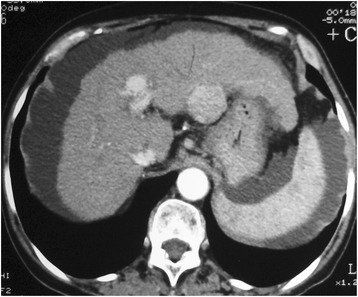
Computed tomography before DEB TACE. Computed tomography showing a hypervascular liver tumor in the left lobe compatible with Hepatocellular Carcinoma in a 71 year-old female patient with liver cirrhosis and hepatitis C

**Fig. 2:**
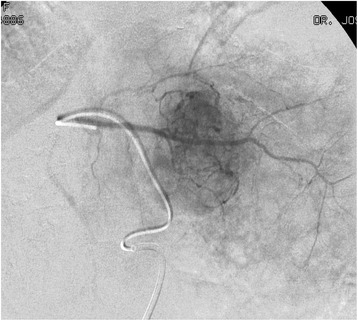
Angiography during DEB TACE. During the DEB TACE procedure the angiography shows the hypervascular lesion

**Fig. 3:**
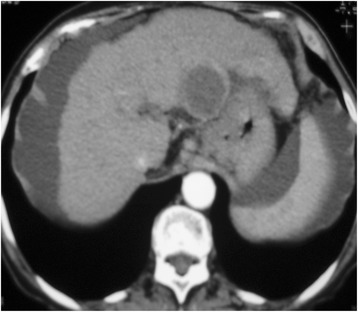
Computed tomography after DEB TACE. Computed tomography 30 days after DEB TACE showing lack of enhancement in the liver tumor consistent with complete response accordingly to the EASL criteria

Procedures, DEB TACE, were done by a staff member of our interventional radiology team with experience with oncology interventions. Two vials of the DEB TACE product DC Beads (2 mL, BioCompatibles Ltd., UK) with a diameter of 100 to 300 μm or 300 to 500 μm were loaded, per vial, with 75 mg of doxorubicin hydrochloride (37,5 mg/mL). Thru the common femoral artery and using a diagnostic catheter (eg. Cobra 5 F) a microcatheter was placed as selective as possible to the vessel irrigating the hepatic tumor. After the tip of the microcatheter achieved a secure point we performed the injection of the DC Beads loaded with doxorubicin mixed with contrast media in a smooth fashion. Our endpoint was to administer the whole two DC Beads vials or when flow of the tumor-nourishing artery reduced markedly. Total stasis of the tumor vascularity was avoided so it wouldn’t disturb the subsequent DEB TACE sessions (Figs. 1, 2 and 3).

### Criteria for therapeutic response

**Fig. 4:**
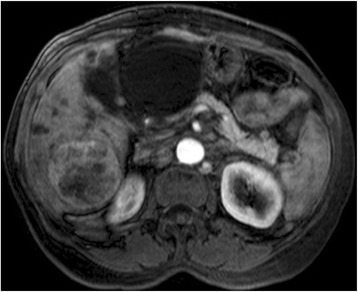
Magnetic Resonance before DEB TACE. A 62 year-old male with alcoholic liver cirrhosis and a large HCC in the right hepatic lobe. At magenetic resonance the lesion is hypervascular with its central portion showing some areas of no contrast enhancement suggestive of necrosis

**Fig. 5:**
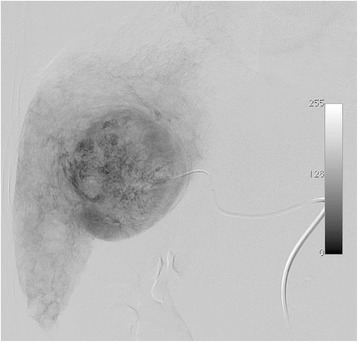
Angiography during DEB TACE. The angiography during TACE shows the large tumor occupying a central position in the liver

**Fig. 6:**
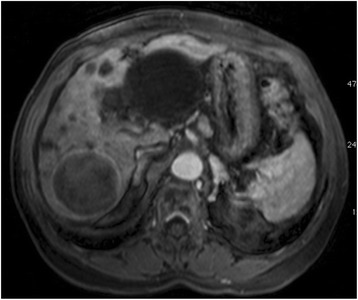
Magnetic resonance after DEB TACE. Magnetic Resonance done four months after DEB TACE showed that the tumor is now avascular. By the EASL criteria there is a complete response but with the Recist criteria the analysis would be just of stable disease

Traditionally the RECIST criteria is used for evaluation of tumor response in solid tumors. According to this criteria it is possible to measure response to treatment by quantifying tumor size reduction and its been validated as a valuable tool in assessing the efficacy of anti-tumor cytotoxic drugs [12]. But strictly anatomical criteria that only take into account the reduction of the size of the tumor to assess response can be misleading when applied to targeted molecular therapies or locoregional treatment (eg. TACE). Frequently HCC liquefies and becomes avascular after a favorable response to TACE, even thought initially it may not show a significant size reduction or no size reduction at all. On this account, in the year 2000, a panel of experts through the European Association for the study of the Liver - EASL [3] - and later in 2008 through the American Association for the Study of Liver Diseases - AASLD [5] - established a series of guidelines for tumor response which included the degree of tumor necrosis for HCC [14]. Therefore, in this current study, the EASL criteria (tumor viability and tumor necrosis) was used. According to this guideline a Partial Response (PR) and Disease Progression (PD) were defined as more than 50% reduction or more than 25% increase, respectively, in the size of the contrast enhancement tumor area of the target lesions. The appearance of new lesions at least 1 cm in size consistent with HCC indicated PD. The enhancement analysis was always performed in the CT or MR contrast arterial [5] phase. An experienced body diagnostic radiologist performed all evaluations (Figs. 4, 5 and 6).

Progression-free survival and overall survival were recorded according to imaging studies and clinical outcomes. Statistical analysis was performed by the Kaplan Meyer method and log-rank using S-plus software. The Kaplan-Meier method was used to describe two outcomes of interest: the probability of survival and the probability of not progressing. The log-rank and Peto test were used to assess whether the survival curves were statistically different between strata of variable categories.

### Assessment of toxicity

Toxicity was assessed after each DEB-TACE session by recording the patient’s clinical status and complete laboratory evaluation. The pain in the immediate postoperative period was registered on a scale of 0 to 10 (0 being no pain and 10 being pain of highest intensity). Adverse events were assessed according to the definitions of the NCI-CTC version 3.0. The occurrence of post-embolization syndrome was also recorded after all treatments [15].

## Results

DEB-TACE was done 52 times in 21 patients. Thirteen patients had BCLC B (62%) HCC and eight patients had BCLC C (38%) HCC with a mean population age of 61 years. Liver cirrhosis was present in 20 patients (95%). The average tumor size was 6.7 cm (range from 3.5 cm to 12 cm). The average number of DEB-TACE procedures per patient was 3 (ranging from 1 to 6 sessions). The mean follow-up was 16.6 months (range 6–30 months). Technical success was achieved in 52 of the 53 DEB-TACE procedures (98%, 52/53). In this single procedure without technical success it was already the fourth DEB-TACE session. During the procedure we identified a complete occlusion of the proper hepatic artery thus preventing the catheter to be placed in suitable position for administration of microspheres loaded with doxorubicin. An average dose of 110 mg (range 75 to 150 mg) of doxorubicin was administered in 52 procedures. The microcatheter was used in all DEB-TACE procedures. The average hospital stay was 1.5 days (range 1–14 days). Only one patient had moderate and persistent pain after DEB-TACE and needed continuous use of analgesics. This patient had partial portal vein thrombosis. We attributed the persistent symptom to the extensive tumor necrosis and wedge shaped areas of suggestive liver parenchyma infarction seen on his MR studies. Overall pain was reported to be minimal to mild (pain intensity score reported averaged at 2.5). Two patients showed hydropic decompensation that were reversed with diuretic therapy. Two patients had increased bilirubin above 3.5 (maximum 4.9), which were also reverted to pre-treatment values in both.

At two years follow-up 12 patients had died. Of those deaths 3 were unrelated to the hepatic cancer (one patient died of acute myocardial infarction, one from a severe pneumonia and a third patient who showed tumor response on imaging studies nevertheless experienced worsening of liver function related to the return of alcohol ingestion). Of the 8 patients who are alive at the 2 years follow-up 7 showed a complete response and 1 patient with partial response. These patients are in clinical and radiological follow-up and no DEB TACE session are scheduled for them in the next 30 days. The patient who had a partial response is currently being evaluated for liver surgery or liver transplant.

### Time to progression and survival

**Fig. 7:**
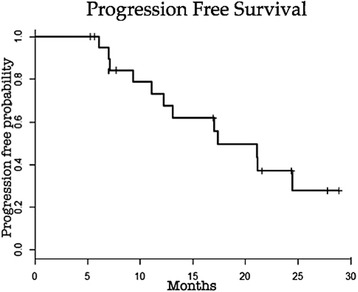
Progression-free survival. Graphic showing Kaplan-Meier estimates of progression-free survival in the 21 patients treated with DEB TACE in our study along the follow-up

**Fig. 8:**
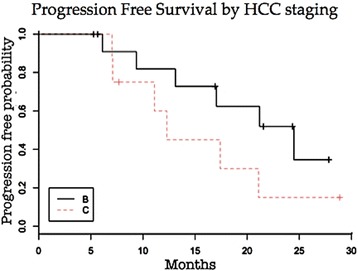
PFS accordingly to HCC staging. Graphic showing Kaplan-Meier estimates of progression-free survival of patients treated with DEB TACE in our study along the follow-up stratified by the HCC BCLC staging classification. Stage B HCC *black line*. Stage C HCC dotted *red line*

The median survival time estimated by the Kaplan-Meier method was 19.6 months. The median time to progression estimated by the Kaplan-Meier procedure was 17.4 months. The progression free and overall survival at 1 and 2 years were 73.0% and 37.1%, 73.7% and 41.6%, respectively (Graphic 1). There was a trend towards a increased survival in patients with BCLC stage B compared to those patients with BCLC stage C (Graphic 2) and patients with lower bilirubin levels (value of bilirubin lower than 2.5), however, without reaching statistical significance (*p =* 0.10 and *p =* 0.11 respectively). Other risks were assessed and showed no association such as gender, race, age, tumor size, portal invasion, alpha-feto protein (AFP) levels, Child-Pugh classification, PS and number of DEB TACE sessions performed (Figs. 7 and 8).

### Security

On average, the toxicity was low to moderate, with a small frequency of grade 2 events (CTCAE v3.0). No Grade 3 or 4 events were reported. Post-embolization syndrome [15] occurred in approximately 50% of patients and was mainly characterized by low-grade fever (up to 38 °C) and malaise lasting an average of 15 days after DEB TACE. In all cases treatments were directed to the symptoms reported and successfully controlled. The laboratory parameters of liver function were not significantly altered after most of the procedures (eg. Liver enzymes usually up to 3 times baseline). No patient died within the first thirty days after DEB TACE. Two patients died within 60 days after the procedure. One of them was discharged 3 days after the procedure with well-compensated liver disease. He was re-admitted 39 days later with liver failure due to worsening of hepatic cirrhosis also associated to the return ethyl derivatives consumption. He remained hospitalized for 21 days and died of progressive worsening of liver function and multiple organ failure. The other patient was discharged 24 h after the procedure. Re-hospitalized 50 days after DEB TACE due to a severe lung infection and died within 7 days.

### Alpha-fetoprotein values

Only 7 patients had augmentation of AFP leves above 200 ng/ml (median 667, range 335–1500). We were able to identify a reduction of approximately 80% in AFP levels after DEB TACE sessions, falling to an average of 133 ng/ml overall. There was a tendency of positive correlation in the reduction of AFP levels and tumor response identified by MR.

## Discussion

TACE is one of the principal medical managements for HCC, being responsible for the treatment of nearly half of all patients with this liver cancer at some point during their disease course [13]. TACE has a limited ability to maintain the chemotherapeutic agent in the liver tumor vascularization. Besides that conventional TACE was the first treatment to show survival benefit in BCLC stage B HCC [4]. Nonetheless it is known that in this conventional approach a chemotherapy blood peak occurs immediately after TACE mainly due to the inability of its well-established vector, lipiodol, to slowly release the drug [14]. The use of embolization material is (eg. Gelfoam particles, PVA) associated with chemotherapy and lipiodol to obtain an acceptable degree of response, often causing a not inconsiderable damage to adjacent non-tumor liver parenchyma speeding deterioration of liver function and increasing the toxicity of this therapeutic modality.

DEB TACE represents an approach to perform chemoembolization employing the administration of microspheres loaded with chemotherapy [17]. Once administered through the catheter, these microspheres in addition to obstruction blood flow also release chemotherapy into tumor vascularity in a controlled and sustained manner for 14 days [18]. This sustained release allows greater exposure of the chemotherapy to cancer cells and thus increasing the degree of tumor necrosis. Furthermore since it is possible to obtain a lower peak of the drug immediately in the systemic circulation after the procedure DEB TACE also appears to cause lower toxicity than the traditional method [14]. With this decrease in morbidity TACE can be repeated more often and thus might have the potential to treat more severe patients who would normally be excluded from conventional TACE (eg. patients with advanced HCC).

One of the feared complications during TACE is inadvertent embolization of non-target organs such as the stomach, gallbladder or the pancreas. One patient reported grade 2 abdominal pain in the right upper quadrant immediately after the completion of DEB TACE and this was attributed to the possible reflux of microspheres into the cystic artery causing acute cholecystitis. This patient was treated symptomatically with no surgical intervention and evolved with resolution of pain in 24 h. During our study the immediate postoperative pain reported was minimal to mild discomfort (pain intensity score reported average was 2.5). Post-embolization syndrome was reported in 50% of our patients. This syndrome is defined as pain, fever, nausea, vomiting and leukocytosis and may occur in up to 90% of patients treated with traditional TACE. Commonly patients do not present all symptoms and they occur in varying intensities [16, 19, 20]. While most patients who present this syndrome are successfully treated with medications directed to the symptoms, this is a complication that in some cases may prolong hospitalization and increase procedure related morbidity. In our study, DEB TACE was extremely well tolerated and all post-embolization syndromes identified were successfully managed in an outpatient approach. In conventional TACE it is not unusual to require powerful painkillers, sometimes narcotics in the postoperative period. In this study the pain reported by patients in the postoperative period was minimal, with the highest intensity observed in the first patient (pain graduated 4/10), which was controlled within 24 h. These findings are consistent with the PRECISION V study [14], a randomized multicenter trial, which compared patients undergoing conventional TACE and DEB TACE. This study showed a significantly lower rate of post-embolization syndrome in patients treated with this new technique [14].

DEB TACE also appears to promote greater tumor response rate. The RECIST criteria, currently used to evaluate tumor response, is based solely in tumor size changes [21]. Criteria such as those from the EASL take into account not only changes in the size of the lesion but also modification in tumor enhancement [22]. The concept is that tumor necrosis is not always accompanied by tumor shrinkage nevertheless it is nearly always followed by reduction on tumor enhancement at contrasted imaging studies such as MR and CT. If we had used the RECIST criteria in our study we would’ve achieved lower response rates which probably would not correspond to the actual therapeutic outcome evaluation. Indeed, not rarely, absence of tumor contrast enhancement after TACE is seen with little or no reduction in the tumor size. This finding was present in 4 of 7 patients who achieved complete response in our study. Moreover, in our study tumor shrinkage was only attested after the second MR imaging follow-up corroborating that tumor response to TACE may not be accompanied by reduction in the size of the liver tumor.

Not infrequently patients responding well to TACE can become resectable or be included in transplantation list due to the occurrence of *downstaging*. In our study, three patients who showed complete response also had reduction of the tumor to less than five cm and thus were evaluated to be included in the liver transplantation list (according to the Brazilian legislation patients with HCC less or equal to 5 cm up to 69 years old may be listed for liver transplantation queue). However in our study, these three patients were over 69 years old and were not listed. One of these patients was also judged to be surgical candidate after evaluation by the Hepato-Biliary surgery team, but due to advanced age (76 years), presence of cirrhosis and some comorbidities surgery was contraindicated. This patient had complete response after 3 sessions of DEB TACE, has been monitored for 30 months with no signs of recurrence or new lesions.

Most of the patients (86%) that presented with an AFP above 200 ng/ml before DEB TACE (*n =* 7, mean AFP of 667, ranging from 335 to 1500) and had radiologic response, also showed a significant decrease on AFP values. The only patient who showed no decrease on AFP value, but had responded according to imaging criteria, also presented bone metastasis after DEB TACE treatment, thus suggesting that the persistently elevated AFP was due to the development of extrahepatic disease.

Time to progression at 1 year and 2 years was 73% and 37.1% respectively and the survival rate at 1 and 2 years was 73.7% and 41.6% respectively. This is similar to the rates published for TACE [23–25]. However, studies evaluating TACE with the new microspheres loaded with chemotherapy, DEB TACE, showed significantly higher survival rates [14, 18]. The lower survival rate in our study could be related to patient selection. Of the 21 treated patients, 8 presented with stage C HCC according to the BCLC criteria. Most studies evaluating DEB TACE and conventional TACE included a significant number of patients with stage A [12, 14, 27]. In addition, in our study, there was a trend of an improved time to progression and survival rate in stage B patients treated with DEB TACE in comparison to stage C patients. Other risks were assessed and showed no association: gender, race, age, tumor size, portal invasion, AFP levels, Child classification, PS and number of DEB TACE sessions performed. According to the BCLC criteria patients with stage C HCC are generally not treated with TACE being referred for treatment with systemic chemotherapy, specifically Sorafenib [8]. This can be a criticism to our study since we didn’t offer the treatment option of Sorafenib for patients with advanced HCC. Patient enrollment happened when Sorafenib was not available for stage C HCC in our institution. Although patients with advanced HCC are usually excluded from TACE because of questionable benefit and unacceptable toxicity, in our group of patients with advanced HCC we didn’t identify any serious adverse event within 30 days after DEB TACE. Of the two patients who died within 60 days after DEB TACE one of them was due to acute myocardial infarction and the other to progression of cirrhosis (patient returned to alcohol consumption and developed rapid deterioration of liver function). These two deaths were not directly related to the procedure performed. Therefore the lower adverse events incidence seen in our stage C HCC patients treated with DEB TACE may indicate that this particular approach might have an acceptable toxicity profile in this population, as been demonstrated in other studies [14, 26, 27].

Although only some few studies evaluated the efficacy of TACE in the treatment of patients with stage C HCC they were able to show benefit in tumor response as well as in survival for patients who underwent TACE when comparing it to supportive treatment [26–28]. In the randomized, multicenter PRECISION V [13] study, which compared conventional TACE with DEB TACE, the subgroup analysis showed that more advanced disease such as Child B and PS 1 patients with tumors in both hepatic lobes or relapsing disease, the incidence of objective response and stable disease was higher, with statistical significance, in patients treated with DEB TACE. The most significant difference was found in the subgroups of patients with PS 1 (Stage C HCC) and Child-Pugh B classification where 63% of patients treated with DEB TACE showed radiological response compared to only 32% of patients treated with conventional TACE [14]. In our study it was also possible to obtain significant tumor response in patients with advanced HCC treated with DEB TACE. Of the 8 patients with advanced HCC, 1 patient had complete response, 4 patients had partial response, 2 patients showed stable disease and 1 patient presented disease progression on imaging studies at 6 months follow-up. 4 patients died at 1 year, 2 patients died at 2 years and 2 patients are still alive on follow-up. In our study, thru the DEB approach it was possible to deliver TACE with low toxicity even to BCLC HCC advanced patients. Nevertheless, because of the inherent higher cost of this treatment we understand that there is not enough evidence to replace conventional TACE for less grave patients or when a not to large liver area is expected to be treated.

## Conclusions

Transarterial chemoembolization with drug-eluting microspheres is able to deliver significant tumor response and progression free survival rate with acceptable toxicity. Larger studies are needed to identify exactly which subset of advanced hepatocellular patients may benefit from this treatment.

## Abbreviations

AASLD: American association for the study of liver diseases
AFP: alpha-feto protein
BCLC: Barcelona clinic liver cancer
CT: Computed tomography
DEB TACE: Drug-eluting beads transarterial chemoembolization
DEB: Drug-eluting beads
EASL: European associantion for the study of the Liver
HCC: Hepatocellular carcinoma
MR: Magnetic ressonance
PD: Disease progression
PR: Partial response
PS: Karnofsky performance status scale
TACE: Transarterial chemoembolization
